# Supplementary material for: Fungal Symbionts Produce Prostaglandin E2 to Promote Their Intestinal Colonization
Source: Front Cell Infect Microbiol. 2019 Oct 18;9:359. doi: 10.3389/fcimb.2019.00359 (PMC6813641; doi:10.3389/fcimb.2019.00359)
Supplement: Supplementary file 1 [file Data_Sheet_1.pdf]

Figure S1

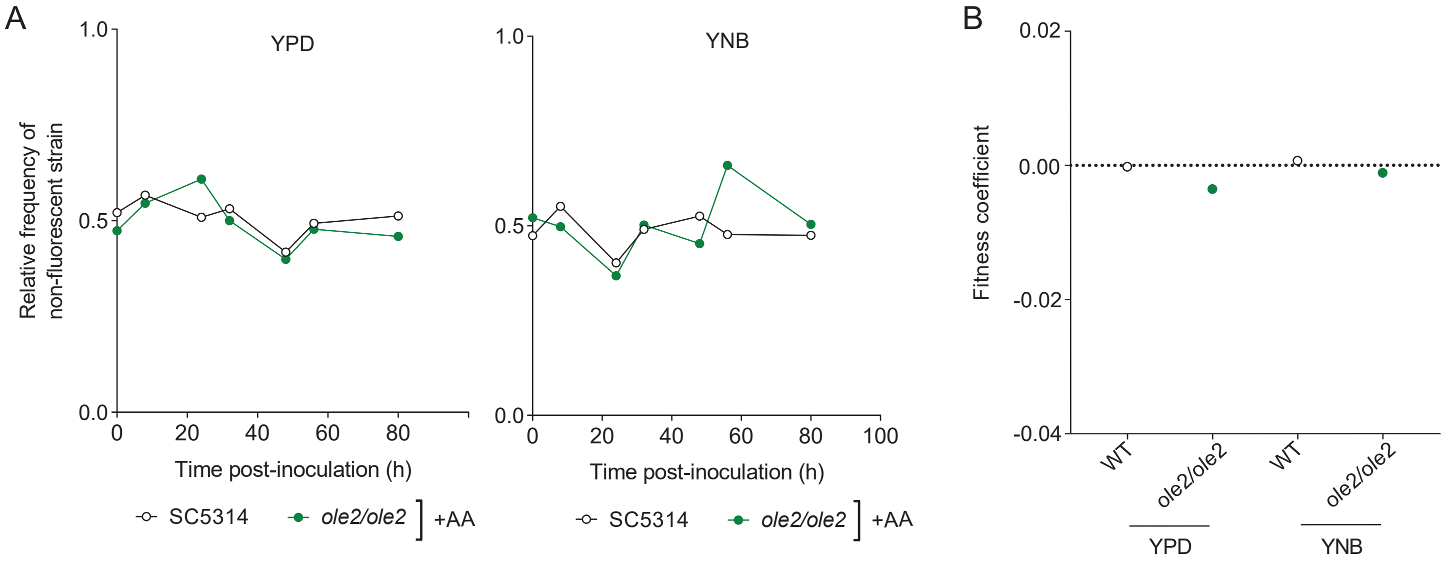

Figure S1. Fungal PGE_2 is not required for the *in vitro* competitive fitness of *C. albicans*.
WT *C. albicans* (SC5314) or the *ole2/ole2* mutant was grown in rich (YPD) or minimal (YNB) media in competition with a fluorescently-tagged WT strain at a 1:1 ratio in serial batch cultures for 80 h in the presence of 0.5 mM AA. (A) Relative frequencies of the WT or *ole2/ole2* strain throughout the serial passages. (B) Fitness coefficients of the WT or *ole2/ole2* strain,determined as outlined in Materials and Methods.

Figure S2

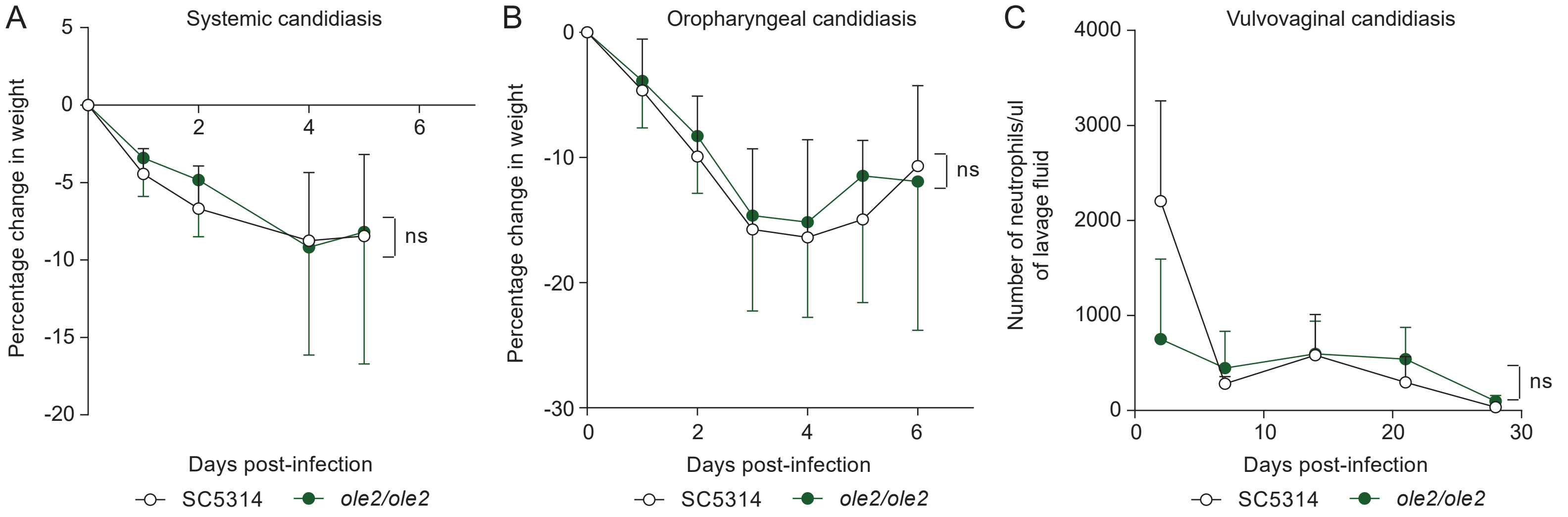

Figure S2. Parameters of *in vivo* virulence of *C. albicans*.
(A-B) Weight change of mice infected systemically (A) or sublingually (B) with the indicated *C. albicans* strains. (C) Neutrophil counts in vaginal lavage fluid over the course of vulvovaginal infection with the indicated fungal strains. Data pooled from 2 independent experiments. Mean +/- s.d.. *n*=8-10 mice/group. Student's *t* test.

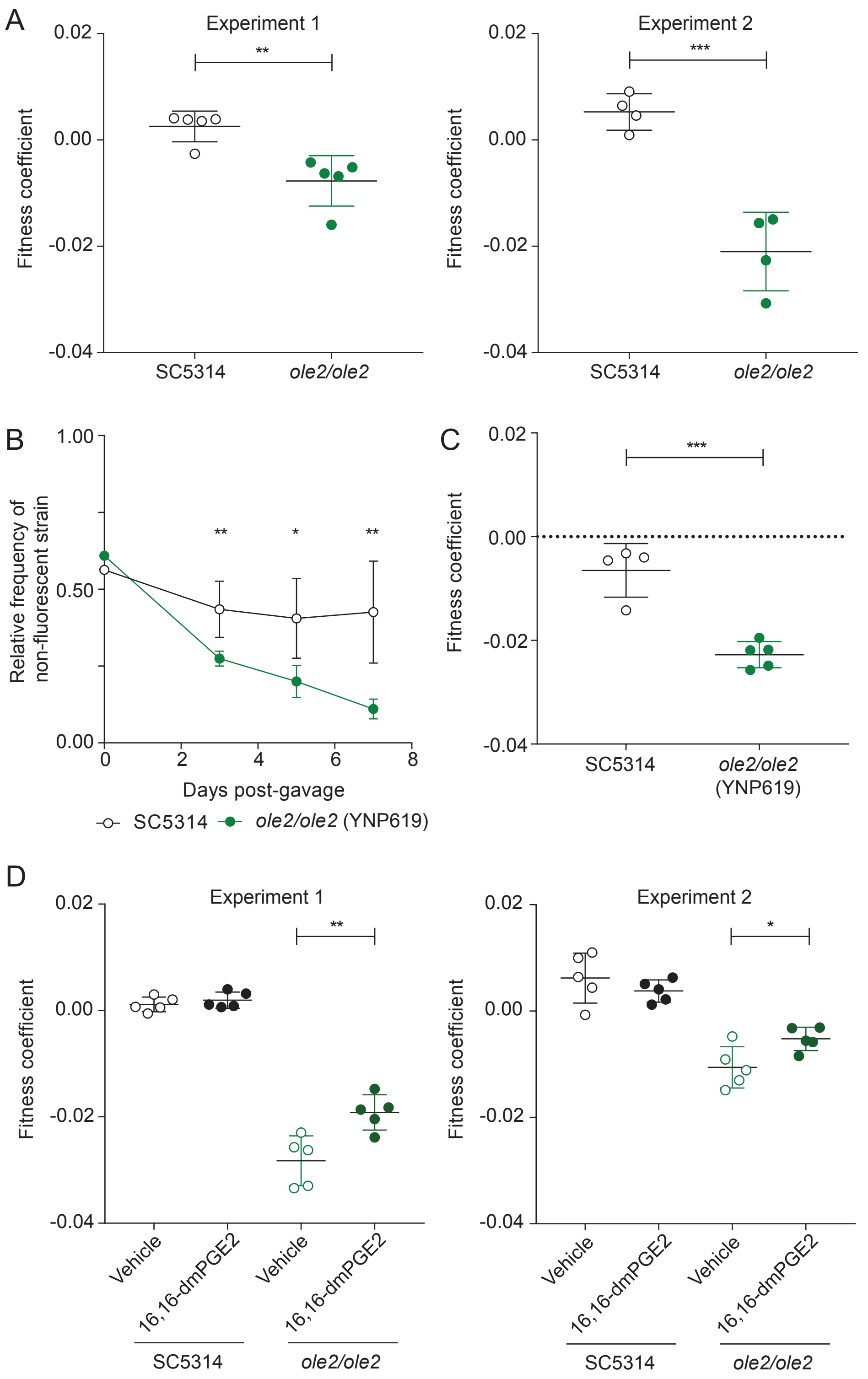

A
Experiment 1
Experiment 2
Fitness coefficient
0.02
0.00
-0.02
-0.04
**
***
SC5314
ole2/ole2
B
Relative frequency of non-fluorescent strain
1.00
0.50
0.00
0
2
4
6
8
Days post-gavage
**
*
**
SC5314
ole2/ole2 (YNP619)
C
Fitness coefficient
***
SC5314
ole2/ole2 (YNP619)
D
Experiment 1
Experiment 2
Fitness coefficient
**
*
Vehicle
16,16-dmPGE2
Vehicle
16,16-dmPGE2
SC5314
ole2/ole2

Figure S3. Fungal PGE_2 promotes intestinal colonization by *C. albicans*.

Mice were inoculated with WT or two independently-derived *ole2/ole2* strains (YNP618 and YNP619) of *C. albicans* in competition with a fluorescently-tagged WT strain as described in Figure 4. (A, D) YNP618 was used. (B-C) YNP619 was used. Mice were left untreated (A-C) or supplemented daily with 16,16-dimethyl-PGE_2 (16,16-dmPGE_2) or its corresponding vehicle (D). Fitness coefficients (A, C-D) and relative frequencies (B) of the indicated fungal strains across independent experiments. Mean +/- s.d.. n=4-5 mice/group per experiment. ***, $p < 0.001$; **, $p < 0.01$; *, $p < 0.05$; Student's t test.

Figure S4

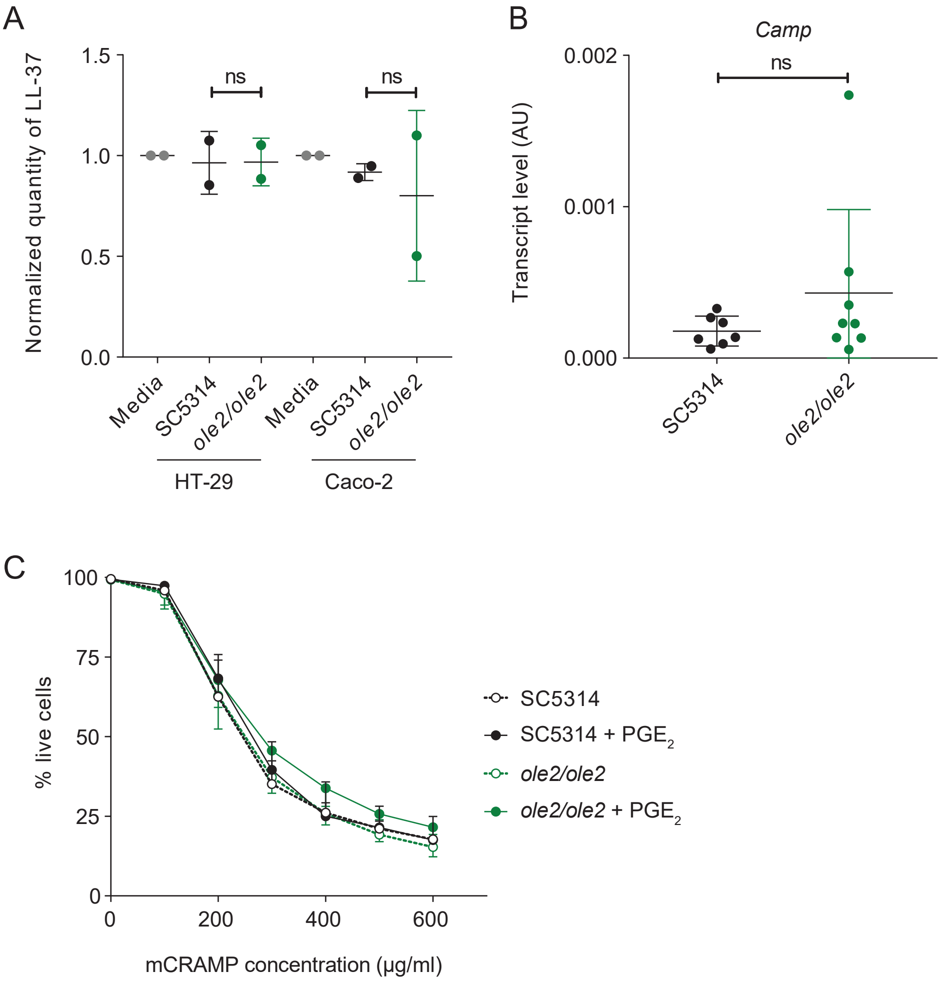

Figure S4. Fungal PGE_2 does not alter host AMP production or fungal resistance to AMPs.

(A) The human IEC cell lines HT-29 and Caco-2 were infected with the indicated *C. albicans* strains at a MOI of 0.5 for 24 h. Secretion of LL-37 was measured by ELISA and normalized to that of uninfected cells (media only). (B) Transcript levels of *Camp* in the colonic tissue of mice colonized with the indicated fungal strains. (C) The viability of the indicated fungal strains after exposure to mCRAMP as assessed by propidium iodide staining. Fungal strains were grown in exponential phase in the presence or absence of PGE_2 prior to incubation with mCRAMP. Data pooled from 2 independent experiments. *n*=6-8 (B). Mean +/- s.d.. ns, not significant. Student's *t* test.

Figure S5

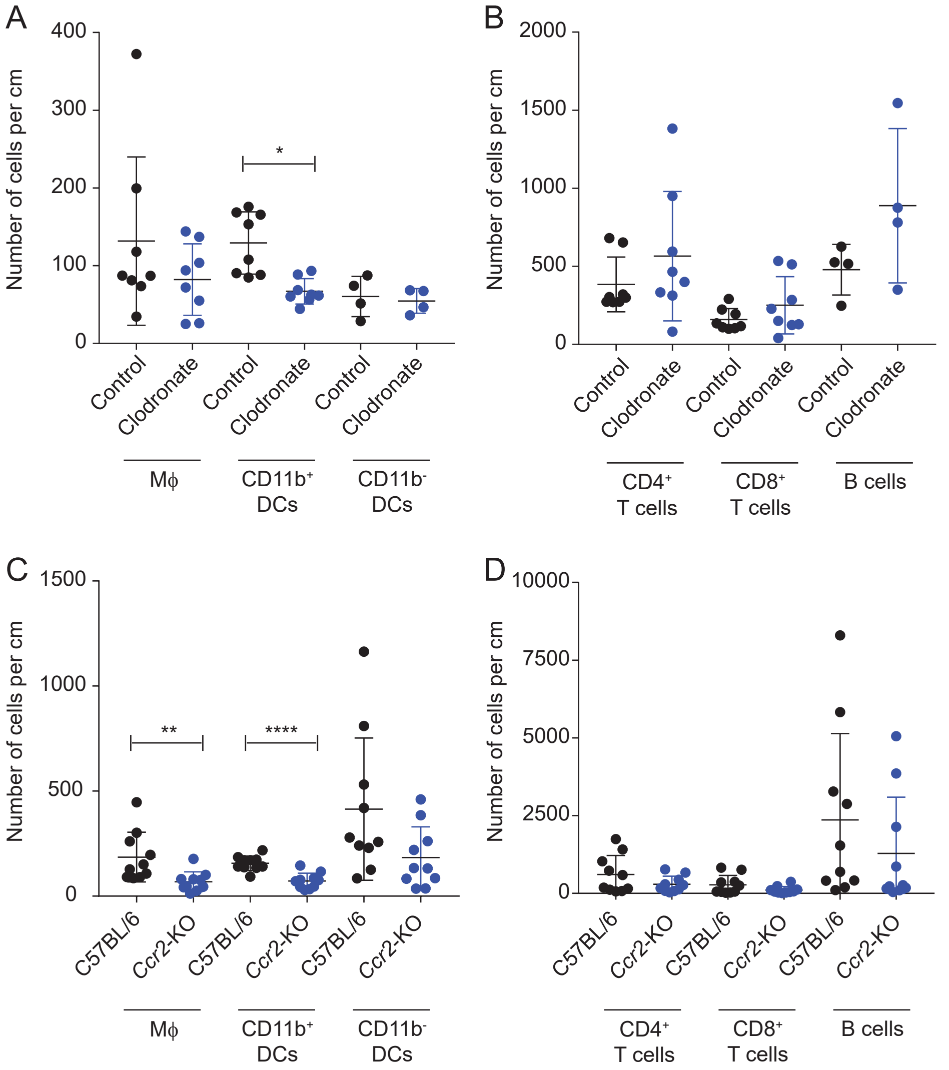

Figure S5. Numbers of various immunocyte populations in the colonic lamina propria of mice depleted of phagocytes 7 days after *C. albicans* gavage.

(A-B) Mice treated with control or clodronate-containing liposomes. (C-D) WT versus *Ccr2*-KO mice. MΦ, macrophage. Data pooled from 2 independent experiments. Mean +/- s.d.. n=4-10 mice/group. ****, $p < 0.0001$; **, $p < 0.01$; *, $p < 0.05$. Student's t test.

Figure S6

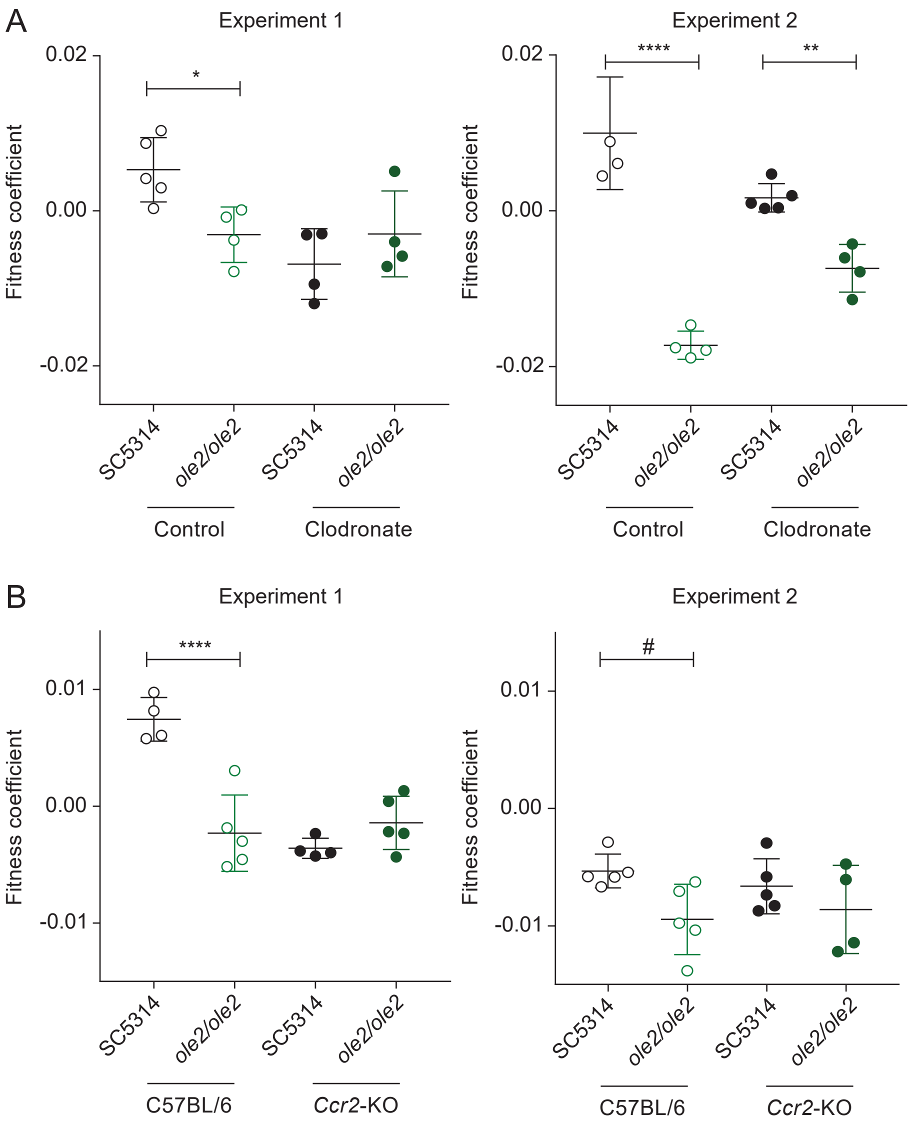

Figure S6. Ablation of colonic phagocytes abrogates the fitness defect of the *ole2/ole2* mutant.

In vivo intestinal colonization experiments were performed as described in Figure 5 and fungal fitness coefficients for each experiment were determined as outlined in Materials and Methods. (A-B) Mice were treated with control or clodronate-containing liposomes. (C-D) WT or *Ccr2*-KO mice were used for intestinal colonization. Data pooled from 2 independent experiments. Mean +/- s.d.. *n*=4-5 mice/group per experiment. ****, $p < 0.0001$; **, $p < 0.01$; *, $p < 0.05$; #, p = 0.059; Two-way ANOVA with Sidak's multiple comparisons test.

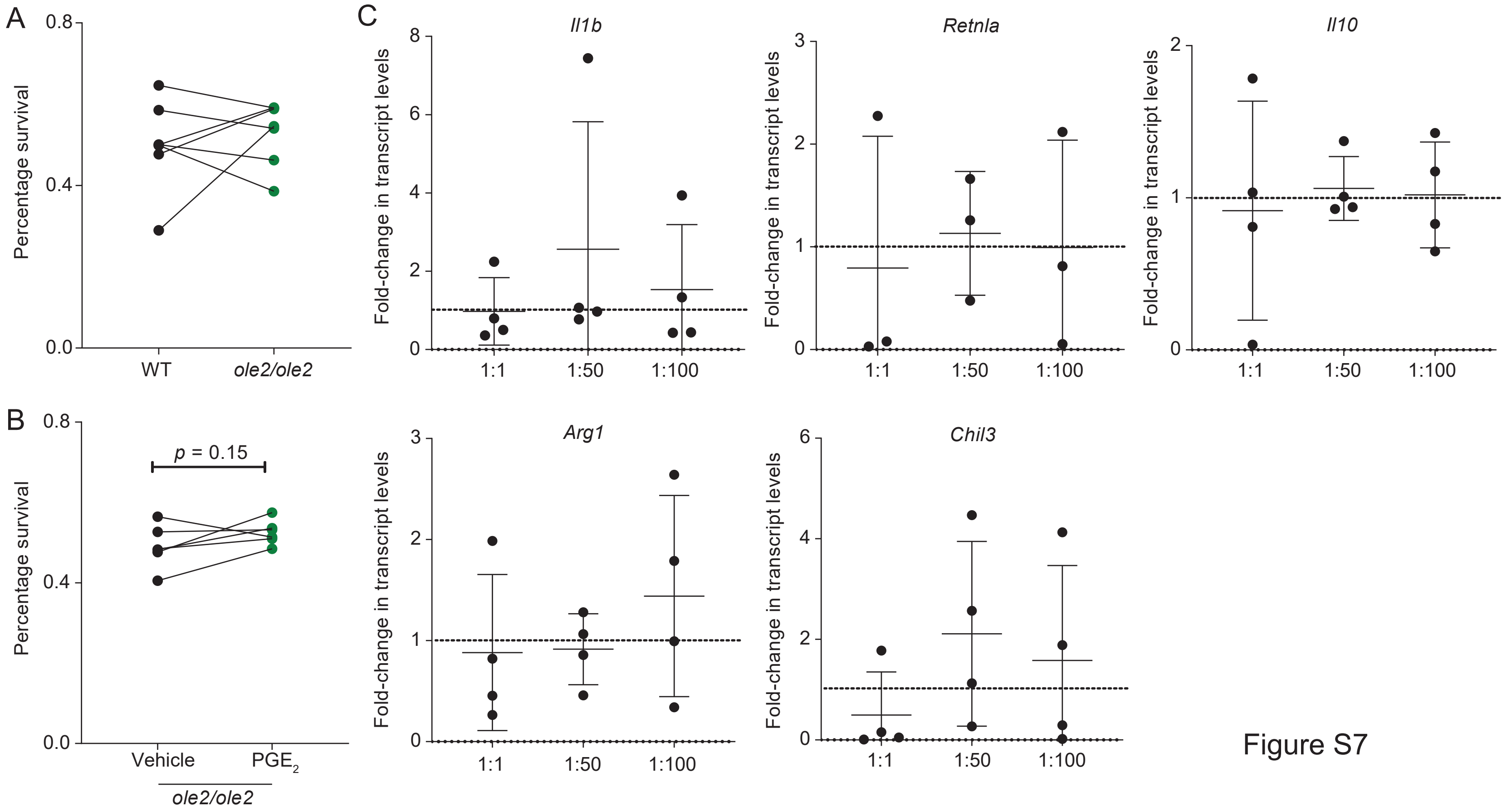
A
B
C
Percentage survival
0.8
0.4
0.0
WT
ole2/ole2
p = 0.15
Vehicle
PGE2
ole2/ole2
Il1b
Retnla
Il10
Arg1
Chil3
Fold-change in transcript levels
1:1
1:50
1:100

Figure S7

Figure S7. Short-term fungal killing by macrophages and transcriptional effects of fungi on macrophages.

(A-B) J774A.1 macrophages were infected with the indicated strains of *C. albicans* and the proportion of surviving fungi within infected macrophages was quantified 3 h post-infection. (B) PGE_2 was added during infection. (C) The transcripts of various pro- and anti-inflammatory cytokines were measured in J774A.1 macrophages infected for 6 h with either the WT or *ole2/ole2* mutant at the indicated MOIs. Data show transcript levels in macrophages infected with the *ole2/ole2* strain normalized to those of macrophages infected with WT *C. albicans*. Each symbol represents one experiment. Mean +/- s.d.. (A-B) Paired student's *t* test. (C) One-sample student's *t* test.

Table S1. Fungal strains used in this study.

Strain ID	Alias	Species	Parental strain	Relevant genotype	Source / Reference
YNP19	SC5314 (WT)	*Candida albicans*		*MAT***a**/**α**	(Fonzi and Irwin, 1993; Odds et al., 2004)
YNP73	SC5314-dTomato	*C. albicans*	YNP19	*eno1::pENO1-dTom-NAT^R/ENO1*	(Sem et al., 2016)
YNP618	*ole2/ole2*	*C. albicans*	YNP19	*ole2Δ::frt/ole2Δ::frt*	This study
YNP619	*ole2/ole2*	*C. albicans*	YNP19	*ole2Δ::frt/ole2Δ::frt*	This study
YNP621	*fet3/fet3*	*C. albicans*	YNP19	*fet3Δ::frt/fet3Δ::frt*	This study
YNP623	*fet31/fet31*	*C. albicans*	YNP19	*fet31Δ::frt/fet31Δ::frt*	This study
YNP3	GA1	*C. parapsilosis*		WT	(Gacser et al., 2007)
YNP69	ATCC 2001	*C. glabrata*		WT	ATCC
YNP92	ATCC 14243	*C. krusei*		WT	ATCC
YNP93	ATCC 13803	*C. tropicalis*		WT	ATCC
YNP94	ATCC MYA-646	*C. dubliniensis*		WT	ATCC
YNP70	BY4741	*Saccharomyces cerevisiae*		*MAT***a** *his3Δ leu2Δ met15Δ ura3Δ*	(Brachmann et al., 1998)
YNP333	972h	*Schizosaccharomyces pombe*		*MAT***a**	S. Oliferenko

Table S2. Primers used in this study. Underlined sequence corresponds to the unique recognition sequence of the restriction enzymes (stated in parentheses) used in this study.

**Primer**	**Sequence (5' → 3')**	**Purpose**
OLE2_UpF1	TTTTTTGGTACCGCCACTGTGTTTTGACAGGC (KpnI)	Mutant construction
OLE2_UpR1	TTTTTTGGGCCCCTGCCCACCATCGAACTGAT (ApaI)	Mutant construction
OLE2_DoF1	TTTTTTCCGCGGGTCAATTGAACTGGGGCACG (SacII)	Mutant construction
OLE2_DoR1	TTTTTTGAGCTCAGCACGTGACTAACCAGGAA (SacI)	Mutant construction
OLE2_rtF1	TGGAACAAGAATTCCCAGAGCA	qPCR
OLE2_rtR1	CCGTGCACTATTGAATCGCC	qPCR
FET3_UpF1	TTTTTTGGTACCTCGTTTCTGTTCACCATTTGTCC (KpnI)	Mutant construction
FET3_UpR1	TTTTTTGGGCCCAGCAGCCGCCAAGAAAACTA (ApaI)	Mutant construction
FET3_DoF1	TTTTTTCCGCGGTGTTGCTGCGTTCTTAGGCT (SacII)	Mutant construction
FET3_DoR1	TTTTTTGAGCTCAGGGGGTTTTCAATAAGGGTGG (SacI)	Mutant construction
FET3_rtF1	ACGCAAATCCTGATGGGGTT	qPCR
FET3_rtR1	TTCTGTGGGATCGGTTCTGC	qPCR
FET31_UpF1	TTTTTTGGTACCAATCCAAACCAAACCAACCACC (KpnI)	Mutant construction
FET31_UpR1	TTTTTTGGGCCCAGCAAGGGGTGGAAAAGTAACA (ApaI)	Mutant construction
FET31_DoF1	TTTTTTCCGCGGTGTTGCACATCAGGTAGGCT (SacII)	Mutant construction
FET31_DoR1	TTTTTTGAGCTCATTAGAGCCGTGGAAAGCCC (SacI)	Mutant construction
FET31_rtF1	TGCACGGTCACGTATTCCAA	qPCR
FET31_rtR1	GCTAAACCTAAGACACCGGCA	qPCR
ACT1_rtF1	TGGAAGCTGCTGGTATTGAC	qPCR
ACT1_rtR1	TTCAGCAATACCTGGGAACA	qPCR

Camp_F1	CTTCAAGGAACAGGGGGTGG	qPCR
Camp_R1	ACCTTTGCGGAGAAGTCCAG	qPCR
Rpl13a_F1	AGGGGCAGGTTCTGGTATTG	qPCR
Rpl13b_R1	TGTTGATGCCTTCACAGCGT	qPCR
Il1b_F1	GCAACTGTTCCTGAACTCAACT	qPCR
Il1b_R1	ATCTTTTGGGGTCCGTCAACT	qPCR
Arg1_F1	CTCCAAGCCAAAGTCCTTAGAG	qPCR
Arg1_R1	AGGAGCTGTCATTAGGGACATC	qPCR
Chil3_F1	CAGGTCTGGCAATTCTTCTGAA	qPCR
Chil3_R1	GTCTTGCTCATGTGTGTAAGTGA	qPCR
Il10_F1	GCTCTTACTGACTGGCATGAG	qPCR
Il10_R1	CGCAGCTCTAGGAGCATGTG	qPCR
Retnla_F1	CCAATCCAGCTAACTATCCCTCC	qPCR
Retnla_R1	ACCCAGTAGCAGTCATCCCA	qPCR

Supplemental references

Brachmann, C.B., Davies, A., Cost, G.J., Caputo, E., Li, J., Hieter, P., and Boeke, J.D. (1998). Designer deletion strains derived from Saccharomyces cerevisiae S288C: a useful set of strains and plasmids for PCR-mediated gene disruption and other applications. Yeast *14*, 115-132.

Fonzi, W.A., and Irwin, M.Y. (1993). Isogenic strain construction and gene mapping in Candida albicans. Genetics *134*, 717-728.

Gacser, A., Trofa, D., Schafer, W., and Nosanchuk, J.D. (2007). Targeted gene deletion in Candida parapsilosis demonstrates the role of secreted lipase in virulence. J Clin Invest *117*, 3049-3058.

Odds, F.C., Brown, A.J., and Gow, N.A. (2004). Candida albicans genome sequence: a platform for genomics in the absence of genetics. Genome Biol *5*, 230.

Sem, X., Le, G.T., Tan, A.S., Tso, G., Yurieva, M., Liao, W.W., Lum, J., Srinivasan, K.G., Poidinger, M., Zolezzi, F.*, et al.* (2016). beta-glucan Exposure on the Fungal Cell Wall Tightly Correlates with Competitive Fitness of Candida Species in the Mouse Gastrointestinal Tract. Front Cell Infect Microbiol *6*, 186.
